# Implementation of a digital health tool for seizure video transfer in a pediatric epilepsy center: A prospective pilot study

**DOI:** 10.1016/j.ebr.2025.100811

**Published:** 2025-07-30

**Authors:** Rahel M. Burger, Gadi Miron, Pascal Fenske, Cornelia Potratz, Angela M. Kaindl, Christian Meisel

**Affiliations:** a Charité-Universitätsmedizin Berlin, Department of Pediatric Neurology and Center for Chronically Sick Children, Berlin, Germany; b Charité-Universitätsmedizin Berlin, German Epilepsy Center for Children and Adolescents, Berlin, Germany; c Charité-Universitätsmedizin Berlin, Charité Pediatric Head & Neck Center, Berlin, Germany; d German Center for Child and Adolescent Health, Berlin Site, Germany; e Computational Neurology, Department of Neurology, Charité – Universitätsmedizin Berlin, Berlin, Germany; f Computational Neurology, Berlin Institute of Health, Berlin, Germany

**Keywords:** Pediatric epilepsy, Digital health, Epilepsy diagnosis, Video messaging

## Abstract

Home videos are increasingly presented in clinical practice, and secure digital tools for clinical video sharing are urgently needed. This study evaluated the feasibility of a secure video-sharing digital platform in pediatric epilepsy diagnostics. We conducted a prospective pilot study that included 60 children (median age 5.5 years) with suspected seizures and their caregivers at a tertiary pediatric center. Participants were invited to submit videos of suspected events through a secure smartphone application. Videos were reviewed by an epileptologist and in parallel participants underwent standard diagnostic evaluation. Outcome measures included video quality, concordance of video assessment with diagnostic workup, and user evaluation questionnaires. Of recruited participants, 16 (26.7 %) used the app to submit 81 videos (median 5.1 videos per patient), with 94 % (76/81 videos) having sufficient quality for meaningful clinical review. Among these participants, 11 (68.8 %) had confirmed epilepsy as their final diagnosis, while 5 (31.2 %) did not. Expert review classified 48.1 % of videos as likely epileptic events, with generalized tonic-clonic seizures being most common (24.7 %). Concordance between submitted videos and final clinical diagnosis was demonstrated in 68.8 % of cases, with higher concordance significantly related to the number of videos submitted (mean 6.4 vs. 2.2 videos per patient, p = 0.04). User evaluation showed favorable ratings for app usability (mean score 5.4/7). This study demonstrates the feasibility of secure digital video transfer in pediatric epilepsy diagnostics, emphasizing the importance of multiple video submissions for accurate diagnosis. Future implementation should address barriers to wider adoption and investigate clinical impact.

## Highlights

• Demonstrates feasibility of secure digital home video transfer in pediatric epilepsy.
• Emphasizes the importance of multiple video submissions for accurate diagnosis.
• Highlights need to address barriers to wider adoption and investigate clinical impact.

## 1 Introduction

Epilepsy is a clinical diagnosis, based primarily on descriptions of events suspected to be seizures, alongside additional information such as EEG findings or clinical observation [1]. Physicians rely on patients or caregivers to provide accurate descriptions of these events, which are known to have relatively low reliability [2]. The differential diagnosis of seizures is broad, and diagnostic inaccuracy is common [3,4], leading to delays in diagnosis in up to 70 % of patients [3]. Unrecognized seizures are the primary cause of delayed diagnosis. These diagnostic delays are particularly concerning in early-onset epilepsy [5], where early recognition of seizures is crucial since delayed diagnosis is associated with worse developmental outcomes, most notably in conditions like infantile spasms [6,7].

The gold standard for seizure and epilepsy classification is video-EEG monitoring (VEM), which is recommended for use in cases of diagnostic uncertainty [8]. However, access to VEM is often limited, with long waiting times at many centres. Additionally, VEM might not be a practical option for patients with infrequent events, since 30 % of patients undergoing VEM do not exhibit seizures during testing [9,10]. With the widespread availability of smartphone cameras, the use of home videos has emerged as a valuable complementary tool for seizure workup and epilepsy diagnosis [[11], [12], [13], [14], [15]]. The review of home videos has been shown to improve diagnostic accuracy when combined with patient descriptions, demonstrate high diagnostic concordance with VEM findings in selected cases, and improve lead time to diagnosis of infantile spasms [12,[15], [16], [17]]. Multiple smartphone apps like Seizure Tracker, Nile and more currently exist, demonstrating the feasibility of improving patient-physician communication using digital health tools [18,19]. The potential benefits of smartphone video for pediatric epilepsy diagnosis have been recently demonstrated by vCreate, a video sharing platform that has significantly shortened diagnostic time and reduced healthcare costs across Scotland and the United Kingdom [20,21].

The German Healthcare System is actively pursuing digitalization through initiatives such as the 2019 German Digital Healthcare Act and the Digital Health Application program (DiGA) [22]. Despite these advances, there are currently no prescribed digital health tools for clinical video sharing in epilepsy care. Consequently, physicians and patients often resort to using email, messaging apps, or other methods that may not comply with medical privacy standards. This study evaluates the feasibility of a secure digital health tool designed specifically for sharing videos of suspected seizures between caregivers and physicians in a tertiary German epilepsy centre. We aimed to assess both the technical implementation and the potential clinical utility of this approach in improving epilepsy diagnosis.

## 2 Materials and methods

### 2.1 Study design and population

**Fig. 1:**
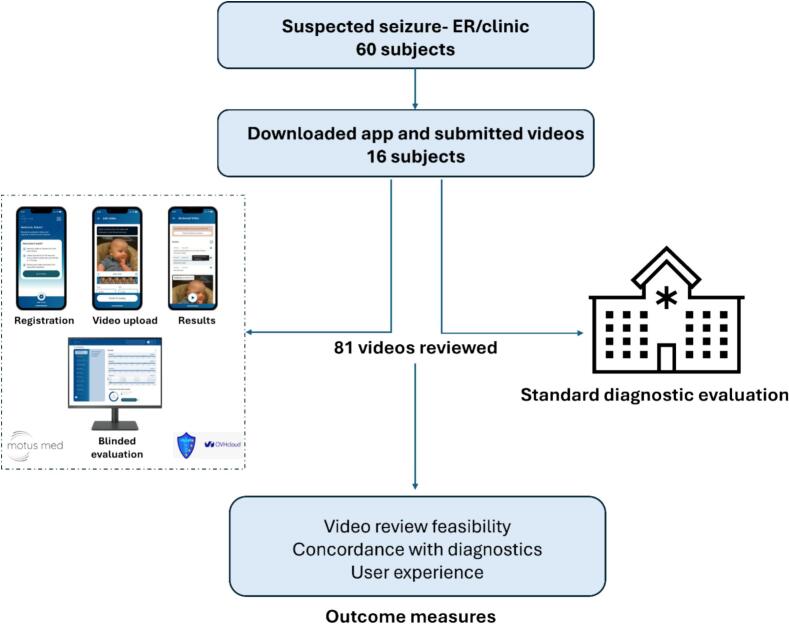
Study design. Participants with a suspected seizure event were given a dedicated digital health app to record and share videos for expert review. In parallel, all subjects underwent standard diagnostic evaluation. The feasibility of video review using the app was evaluated, as well as the concordance of video reviews with the final diagnosis and the overall user experience.

We conducted a prospective observational study of pediatric patients with suspected seizures between February 2024 and June 2024 at the Department of Pediatric Neurology at Charité − Universitätsmedizin Berlin, a tertiary pediatric neurology center in Germany. The overall study design is illustrated in Fig. 1.

Children and adolescents presenting with events suspicious for seizures, who had caregivers with a smartphone that were willing to record events were eligible participants. Exclusion criteria included inability to provide informed consent, inability to use the smartphone application, and German or English language proficiency inadequate to properly understand the study information. Participants were recruited from the emergency department and outpatient clinic during visits for suspected seizure disorders. Upon enrollment, all caregivers received a standardized, brief in-person orientation, including demonstration of app functions and guidance on video capture. They were encouraged to record any events they considered unusual or concerning. Caregivers used the app in parallel with and until the completion of the standard diagnostic workup. videos were uploaded from the app to a secure cloud and displayed to doctors via a dedicated web-based platform. All videos were reviewed by an expert pediatric epileptologist (CP) who was blinded to the results of clinical workup to prevent bias in video classification. After video review, caregivers received notification of the classification results through the app.

The primary outcome measures were: [1] feasibility, assessed by the technical quality of submitted videos, defined as videos with sufficient clarity, duration, and field of view to enable clinical interpretation; and [2] user satisfaction with the digital tool as measured by the German language mHealth App Usability Questionnaire [23,24]. Secondary outcomes included diagnostic concordance between video-based classifications and final clinical diagnoses of the events, the distribution of seizure types captured, and identification of factors associated with successful video capture.

For all participants, we collected comprehensive clinical and demographic information including age, sex, clinical presentation, medical history, seizure semiology, concurrent medications, and final diagnosis following standard clinical evaluation.

### 2.2 Video collection and review

To facilitate secure video sharing between families and clinicians, we developed a digital health tool consisting of two components: [1] a user-friendly smartphone application, motus *med*, for caregivers and [2] a web-based review platform for clinicians. The smartphone app was designed with a simple interface allowing caregivers to either record videos directly through the app or upload existing videos from their device gallery. Users could perform basic editing functions such as trimming video length before securely transmitting recordings to the clinical team.

On the physician side, the web-based platform enabled structured review and classification of submitted videos. Each video was classified into one of three categories: [1] seizure, [2] non seizure movement/behaviour, or [3] unclear/indeterminate. For videos classified as seizures, the reviewer further specified the seizure type according to the International League Against Epilepsy (ILAE) classification system and documented key semiological features when visible.

Both components of the digital tool operated on a secure, medical-grade cloud infrastructure (OVH) with servers located exclusively in Germany, ensuring compliance with European data protection regulations (GDPR) and German healthcare privacy standards. Access to video content was strictly limited to authorized study physicians, and all data transmission was encrypted using industry-standard protocols.

### 2.3 Statistical analysis

Descriptive statistics were used to characterize the study population and video submissions. Continuous variables are presented as means with standard deviations or medians with ranges, as appropriate based on data distribution. Categorical variables are presented as counts and percentages. To evaluate differences in video submission patterns between participants with epilepsy versus non-epileptic diagnoses, the Mann-Whitney *U* test was applied due to the non-normal distribution of the data.

For assessing diagnostic concordance, we compared the classification of videos by the reviewing pediatric neurologist with the final clinical diagnosis regarding the events in question, as were established through standard clinical evaluation (including neurological examination, EEG, and neuroimaging when indicated). We then explored factors associated with diagnostic concordance, including the number of videos submitted per participant.

### 2.4 Evaluation of usability

To assess user experience and acceptance of the digital health tool, we employed the validated German mHealth App Usability Questionnaire Short (G-MAUQ-S). All study participants who submitted at least one video were invited to complete the questionnaire after using the application. The questionnaire consisted of six items rated on a 7-point Likert scale (1 = strongly disagree to 7 = strongly agree): (1) The app was easy to use; (2) It was easy for me to learn to use the app; (3 I would use this app again.; (4) Overall, I am satisfied with this app.; (5) The app would be useful for my health and well-being; and (6) The app helped me manage my health effectively.

### 2.5 Eathics statement

The study was approved by the Institutional Review Board at Charité – Universitätsmedizin Berlin (ethics application number 2/273/23). Informed consent was obtained from all parents/guardians, and assent was obtained from children over 7 years of age when appropriate.

## 3 Results

### 3.1 Study population

Of 60 participants recruited for the study, 16 (26.7 %) used the app to upload a total of 81 video recordings. The median age of these participants was 5.5 years (range: 1.4–17.2 years), with nine males (56.3 %). Eleven participants (68.8 %) had confirmed epilepsy, while 5 (31.2 %) did not as a final diagnosis. Detailed demographic and clinical characteristics are presented in Table 1, Table 2.

**Table 1:** Clinical and demographic characteristics of participants.

Characteristic	Value
Total participants	16
Age, median (range), years	6.3 (1.4–17.2)
0–2 years, n (%)	5 (31.3)
3–6 years, n (%)	6 (37.5)
7–12 years, n (%)	3 (18.8)
13–17 years, n (%)	2 (12.5)

Sex, n (%)	
Male	8 (50.0)
Female	8 (50.0)
Presentation setting, n (%)	
Emergency Room	5 (31.3)
Social Pediatric Center	11 (68.8)
Diagnostic category, n (%)	
Epilepsy	11(68.8)
Non-epileptic disorders	5 (31.3)

Videos submitted	
Total videos	81
Videos per participant, mean (range)	5.1 (1–19)
Videos from epilepsy patients, mean (range)	6.2 (1–19)
Videos from non-epilepsy patients, mean (range)	3.0 (1–4)

**Table 2:** Clinical details of diagnostic workup.

ID	Age, years	Sex	Reason for Presentation	Event Frequency	EEG Findings	MRI Findings	Diagnosis	Anti-seizure medication	Comorbidities	# Videos submitted	Video Reviewed as Seizure/Non-seizure/Unclear
1	11. 4	F	Unresponsiveness episodes	Daily	Frontoparietal right spike-wave discharges	−	Focal epilepsy of unknown origin with hypermotor seizures	1	−	3	0/0/3
2	2	F	Paroxysmal movements of UE	Daily	−	−	Non epileptic infantile stereotypies	−	Developmental delay	2	0/0/2
3	6.3	M	Cyanotic episodes	Weekly	Generalized spike wave discharges	Medullary gliosis, Parenchymal hypoplasia	Non epileptic events	1	Prematurity	3	0/0/3
4	1.5	F	Suspected epileptic spasms	Daily	−	−	Non epileptic infantile stereotypies	−	−	3	1/1/1
5	1.4	F	Paroxysmal movements of UE	Daily	−	−	Non epileptic breath-holding spells	−	−	4	2/0/2
6	2.6	F	Cyanotic episodes	Daily	Focal seizure with right parietal onset	−	Generalized epilepsy with tonic-clonic seizures	1	Breath-holding spells	19	7/10/2
7	4.4	M	Teeth grinding and laughter episodes	Daily	−	Reduced myelination, corpus collosum hypoplasia	Atypical Rett-syndrome (*FOXG1*)	DRE	Dystonia	7	2/5/0
8	7.9	M	Suspected epileptic spasms	Daily	Generalized spike and wave discharges	Cerebral manifestation of congenital CMV-infection	Lennox-Gastaut syndrome	DRE	Congenital CMV-infection	4	3/0/1
9	6.5	M	Evaluation of new events	Weekly	Parietooccipital left and occipital right spike and wave discharges	Right parietal lesion	Epilepsy of unknown etiology with gelastic seizures, absences and generalized tonic-clonic seizures	DRE	Pulmonary artery stenosis, pre-excitation syndrome	4	3/0/1
10	11.2	M	Evaluation of new events in patient with known epilepsy	Daily	Focal seizure with left temporal parietal onset	Suspected FCD L parietal	Epilepsy of structural origin with focal onset and secondary generalized tonic seizures	DRE	−	6	4/0/2
11	2.7	M	Suspected epileptic spasms	Daily	Hypsarrhythmia	Hippocampal tumor	Epilepsy of structural origin with epileptic spasms	2	−	8	8/0/0
12	6.4	M	Activity arrest episodes	Monthly	Fronto- temporal right spike and wave discharge	Unspecific white matter changes	Epilepsy of structural origin with activity arrest and gaze rigidity	DRE	CP	1	0/1/0
13	4.8	F	Tonic upgaze with paroxysmal movements of UE and LE	−	Generalized spike and wave discharges	Lissencephaly, dysplastic BG/thalami, brainstem atrophy	Developmental and epileptic encephalopathy	DRE	Miller-Dieker-Syndrome	12	8/1/3
14	3.8	F	Gaze deviations	Hourly	Irregular posterior spike and wave discharges	−	Eyelid myoclonia with and without absences (Jeavons)	1	Long QT syndrome	1	0/1/0
15	17.2	F	Paroxysmal movements	Monthly	−	PVL	Non epileptic behavioral events	−	Prematurity	1	0/1/0
16	15	M	Paroxysmal movements	Daily	−	−	Epilepsy of unknown origin with myoclonic seizures	1	Autism, developmental delay	3	1/0/2

Table includes all participants in the study. For participants with a known diagnosis of epilepsy, the reason for presentation describes new events that required diagnostic workup. Abbreviations: EEG – electroencephalographic; MRI- magnetic resonance imaging; UE – upper extremity; LE – lower extremity;

### 3.2 Seizure classification

Participants submitted a mean of 5.1 videos (range 1–19) per patient. Participants with epilepsy submitted more videos than those with non-epileptic events (mean 6.2 vs. 2.6 videos per participant); however, this difference was not statistically significant (p = 0.1, Mann-Whitney *U* test). The age group 3–6 years contributed the largest number of videos (n = 48, 59.2 % of total), despite representing only 37.5 % of participants.

Expert review classified 39 videos (48.1 %) as epileptic events and 42 (51.9 %) as non-epileptic or uncertain events. Seventy-six videos (93.8 %) had sufficient technical quality for visual review. Among epileptic events, generalized tonic-clonic seizures were most common (20 events, 24.7 % of total), followed by focal motor seizures (10 events, 12.3 %) and epileptic spasms (3 events, 3.7 %). Detailed semiological features were documented in 48.4 % of epileptic events. The distribution of seizure types and most common semiological features are presented in Table 3.

**Table 3:** Classification of videos and semiological features.

Category and Feature	Number	Percentage
Total videos	81	100
Suspected seizures	39	48.1
Generalized tonic-clonic seizures	20	24.7
Focal motor seizures	10	12.3
Behavioral arrest	6	7.4
Epileptic spasms	3	3.7

Common semiological features		
Gaze deviation	4	10.2*
Tonic activity	4	10.2*
Clonic activity	4	10.2*
Oral automatisms	4	10.2*
Myoclonic features	3	7.6*
Non-seizure/Uncertain events	41	50.6
Non-seizure	20	24.7
Uncertain (excluding technical issues)	17	21.0
Technical failure	5	6.2

* Percentage of epileptic events (n = 39).

### 3.3 Concordance with clinical diagnoses

Overall, 68.8 % of participants (11/16) showed concordant results between video classifications and clinical diagnoses. Concordance rates were 72.7 % (8/11) for participants with epilepsy diagnoses and 60 % (3/5) for those with non-epileptic diagnoses. Focal motor seizures, generalized tonic-clonic seizures (GTCS) and epileptic spasms were identified with 100 % concordance in the app. In contrast, seizure types such as activity arrest, myoclonic movements, and hypermotor seizures showed considerably lower concordance rates, ranging from non-concordance to 50 % concordance.

Diagnostic concordance was significantly related to the number of videos submitted, with concordant participants submitting a mean of 6.3 compared to 2.4 videos per non concordant subject (p = 0.04, Mann-Whitney U Test). To note, among subjects with a final diagnosis of epilepsy, only 52.9 % (36 of 68 videos submitted) were of seizures, indicating that even in patients with confirmed epilepsy, not all recorded events represent typical seizures. The remaining videos from epilepsy patients were classified as “Not detected” (18 videos, 26.5 %), “Unclear” (14 videos, 20.6 %).

### 3.4 User evaluation

Ten participants completed the mHealth App Usability Questionnaire (MAUQ) to evaluate the usefulness and usability of the app (Table 4.). The overall mean score across all questions was 5.4 ± 0.4 on a 7-point Likert scale. Usability aspects received the highest ratings, with “The app was easy to use” scoring a mean of 6.0 ± 0.8 and “It was easy for me to learn to use the app” scoring 5.9 ± 1.0). The lowest-rated aspect was “The app helped me manage my health effectively” with a mean score of 4.7 ± 1.9, suggesting that while participants found the app easy to use, some were less confident about its direct impact on health management.

**Table 4:** mHealth App usability Questionnaire (MAUQ) results.

Question	Mean Score ± SD
The app was easy to use	6.0 ± 0.8
It was easy for me to learn to use the app	5.9 ± 1.0
I would use this app again	5.5 ± 1.5
Overall, I am satisfied with this app.	5.4 ± 1.6
The app would be useful for my health and well-being.	5.3 ± 1.4
The app helped me manage my health effectively.	4.7 ± 1.9

Table shows results for the usability questionnaire. Participants answered the questions on a Likert scale between 1–7, with 7 being the highest score.

## 4 Discussion

In this single centre prospective pilot study, we found that it is feasible to use a digital health tool to securely transfer videos of suspected seizures to treating physicians in a tertiary German pediatric epilepsy centre. This study thus represents one of the first attempts to systematically evaluate such technology within the German healthcare system, where digital health tools have not yet become standard practice in epilepsy care despite their potential benefits.

The ability of caregivers to capture diagnostically relevant videos without specialized equipment or extensive training suggests that this approach could effectively extend clinical observation beyond the hospital setting. In our study, 94 % of videos could be interpreted by the treating physician, and in approximately half of the cases with suspected epileptic seizures, localizing semiology was described by the reviewing epileptologist. This suggests that not only do caretakers understand how to take videos of their children, but they can also do so fast enough after the seizure onset. Our findings thus build on prior studies, in different settings, that have shown that the use of home videos in epilepsy is feasible and helpful [[11], [12], [13],[15], [16], [17]]. Despite the technical ease of use, there was a relatively low participation rate of 26 % amongst recruited individuals. This discrepancy suggests that while the technology itself functions well, adoption barriers exist. These included in-hospital connectivity issues, language limitations, potential privacy concerns regarding sharing sensitive video data, and application accessibility (app was only available as a research tool requiring a lengthy installation process, and not through the apple and google public app stores). To address these challenges and ensure equitable access for underserved populations, future implementations should incorporate multilingual support, simplify the installation process, ensure availability in public app stores (e.g. Apple and Google), and include targeted outreach programs to engage a broader user base.

The moderate concordance between video assessment and final clinical diagnoses demonstrates both the promise and limitations of video as a standalone diagnostic tool. The significant association between number of videos submitted and diagnostic concordance, coupled with the finding that less than half of videos from confirmed epilepsy patients captured seizures, highlights the importance of multiple sampling of suspicious events. This suggests that clinical protocols for smartphone video-based digital health tools should emphasize collecting multiple events rather than just a single “representative” episode. Notably, during this study, three subjects with epileptic spasms submitted videos that were correctly identified by the reviewing physician. Since epileptic spasms are brief paroxysmal events that often appear in clusters, digital video tools are particularly well-suited for their capture and identification. This application is especially valuable given the critical importance of early diagnosis and intervention for improved developmental outcomes in epileptic infantile spasm syndrome [[5], [6], [7]].

User evaluation revealed high usability ratings alongside moderate perceived health impact. The strong usability scores align with the excellent technical performance of the app, whereas the more moderate health impact perception likely reflects our study design, which positioned the app as supplementary to the standard diagnostic pathway within an academic neurological centre. This implementation context matters significantly, as perceived value depends heavily on how digital tools are integrated into existing care pathways and is critical for adoption of novel technologies by both physicians and patients [25]. Future implementation should clearly communicate the advantages of video-sharing tools during user recruitment and ensure optimal settings for clinical benefits.

This study has several notable limitations. First, as a pilot study, it was not powered to evaluate clinical benefits of the app and given the limited sample size, analysis was restricted to univariable models. Second, being limited to a single tertiary centre in a major city, findings might not generalize to rural communities or smaller clinical centres. Third, video assessment relied on a highly experienced epileptologist, and concordance rates might be lower with less specialized physicians. Fourth, while the blinding protocol enhanced objectivity, it created an artificial separation between video review and clinical information that would not exist in practice, where videos would be interpreted alongside history, examination findings, and test results. Fifth, the voluntary nature of video submission introduces selection bias, as highly motivated families were likely overrepresented.

Future application of digital tools for video sharing in epilepsy care require thoughtful integration within existing clinical workflows. One key component is secure and compliant digital infrastructure to enable safe video communication at scale between families and providers. Another critical parameter is interoperability with electronic health records, allowing video assessments to become part of the patient medical record. Once these prerequisites are met, the diagnostic potential of this approach could be further enhanced through machine learning algorithms that pre-screen videos or highlight features of interest for clinical review. The field of artificial intelligence for video-based seizure detection shows promising results, with studies demonstrating not only feasibility, but also high performance metrics [[26], [27], [28], [29]]. Of note, recent work has demonstrated that AI models can be used to detect epileptic spasms on smartphone videos with high performance metrics [30]. However, it must be emphasized that video analysis, even when enhanced by technology, complements rather than replaces comprehensive clinical evaluation. The approach should be viewed as accelerating the pathway to appropriate diagnostic evaluation and treatment. Future research should focus on implementation outcomes including changes in time-to-diagnosis, treatment initiation speed, healthcare resource utilization, and most importantly, patient outcomes in diverse clinical settings.

## CRediT authorship contribution statement

**Rahel M. Burger:** Writing – original draft, Visualization, Investigation. **Gadi Miron:** Writing – original draft, Visualization, Formal analysis, Conceptualization. **Pascal Fenske:** Writing – review & editing, Investigation. **Cornelia Potratz:** Writing – review & editing, Investigation. **Angela M. Kaindl:** Writing – review & editing, Investigation. **Christian Meisel:** Writing – original draft, Investigation, Conceptualization.

## Declaration of competing interest

The authors declare the following financial interests/personal relationships which may be considered as potential competing interests: C.M is supported by NeuroCure Cluster of Excellence, funded by the Deutsche Forschungsgemeinschaft (DFG, German Research Foundation) under Germany ´s Excellence Strategy EXC-2049-390688087. Authors R.B, G.M, P.F, C.P have no conflict of interest. A.M.K. declares no conflict of interest with respect to the current manuscript. She has received funding and honoraria from Desitin, Angelini Pharma, Neuraxpharm, Vertex, Nightwatch.
